# Supplementary material for: Finding novel vulnerabilities of hypomorphic BRCA1 alleles
Source: Mol Oncol. 2026 May 29:10.1002/1878-0261.70271. Online ahead of print. doi: 10.1002/1878-0261.70271 (PMC13399126; doi:10.1002/1878-0261.70271)
Supplement: Supplementary file 4 — Fig. S1. Genome‐wide CRISPR‐Cas9 knockout screen preparation. Fig. S2. BRCA1‐depleted cells are sensitive to CSA loss. Fig. S3. NDE1 −/− cells were successfully made and complemented. [file MOL2-9999-0-s002.pdf]

Supplemental figure 1

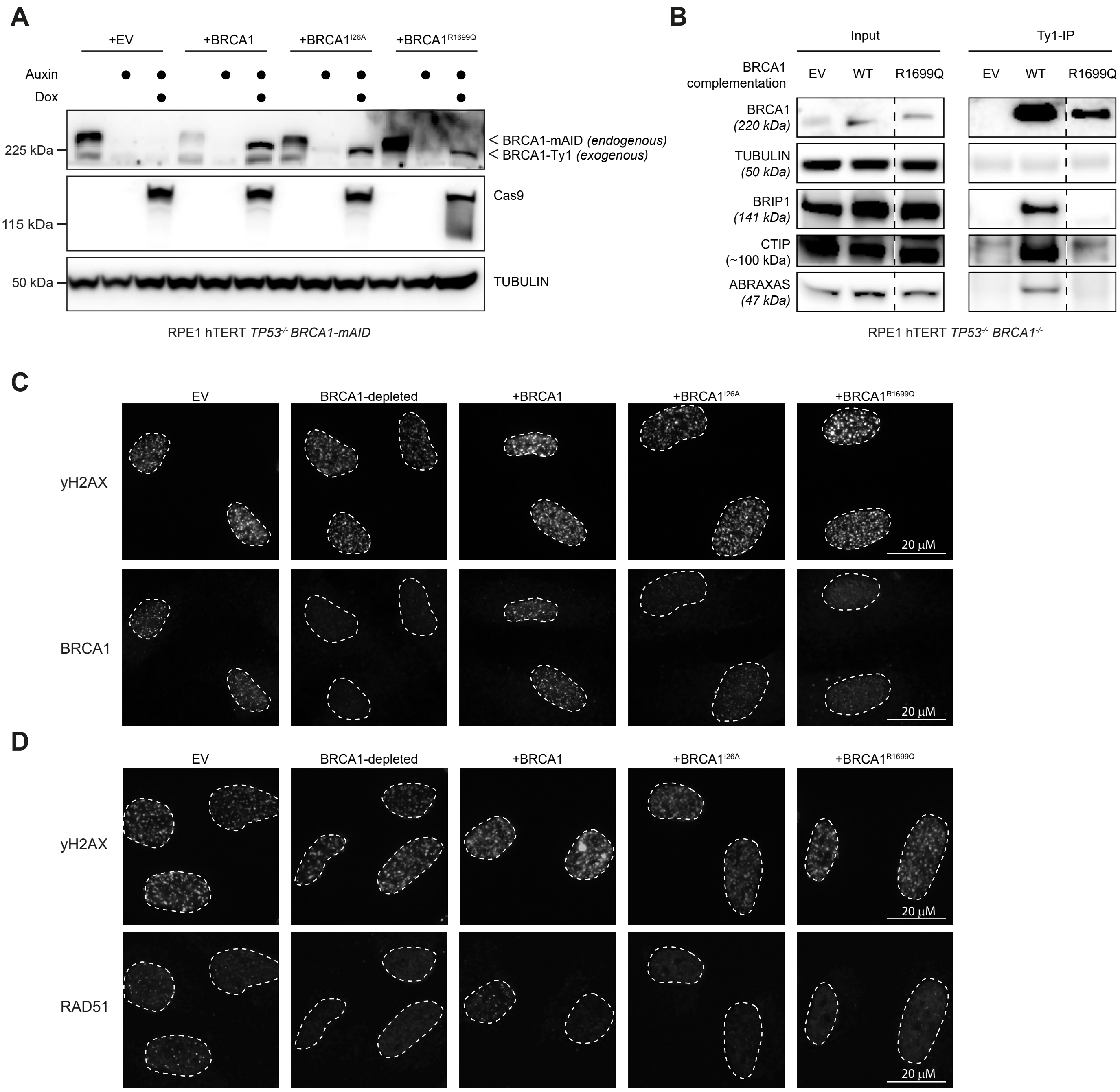

E

	Survival (confluency)
Control	95%
+ sg*PSMD1*	95%
+ sg*PSMD1* + Doxycycline	3%

F

	Doubling time (hours)
BRCA1-mAID (proficient)	28,84
BRCA1-mAID + Auxin (depleted)	42,88
BRCA1-mAID + BRCA1	27,91
BRCA1-mAID + BRCA1^{I26A}	36,12
BRCA1-mAID + BRCA1^{R1699Q}	43,36

Supplemental figure 1. Genome-wide CRISPR-Cas9 knockout screen preparation (A) The expression of BRCA1 and Cas9 was assessed by Western blotting of the indicated complemented RPE1 hTERT *TP53*$^{-/-}$ *BRCA1-mAID* (auxin-inducible degron) + doxycycline inducible Cas9 cells lines. Data shown represent three independent experiments. **(B)** Ty1 immunoprecipitation on RPE1 hTERT *TP53*$^{-/-}$ *BRCA1*$^{-/-}$ cells complemented with BRCA1-Ty1 and BRCA1^{R1699Q}-Ty1. Dashed line indicates removal of non-relevant lanes post-imaging. Data shown represent four independent experiments. **(C)** Representative microscopy images of the experiment in Figure 1A. White line indicates the scale bar, 20 µM (n=2). **(D)** Representative microscopy images of the experiment in Figure 1B. White line indicates the scale bar, 20 µM (n=2). **(E)** The eficiency of virally integrated inducible Cas9 cassette was assessed by survival of the cells after transduction with a sgRNA against *PSMD1*, an essential gene. Data shown represent two independent replicates. **(F)** The growth speed of each indicated RPE1 hTERT *TP53*$^{-/-}$ cell line was assessed by monitoring cell growth and calculating the doubling time. Data shown is the average of two independent replicates.

Supplemental figure 2

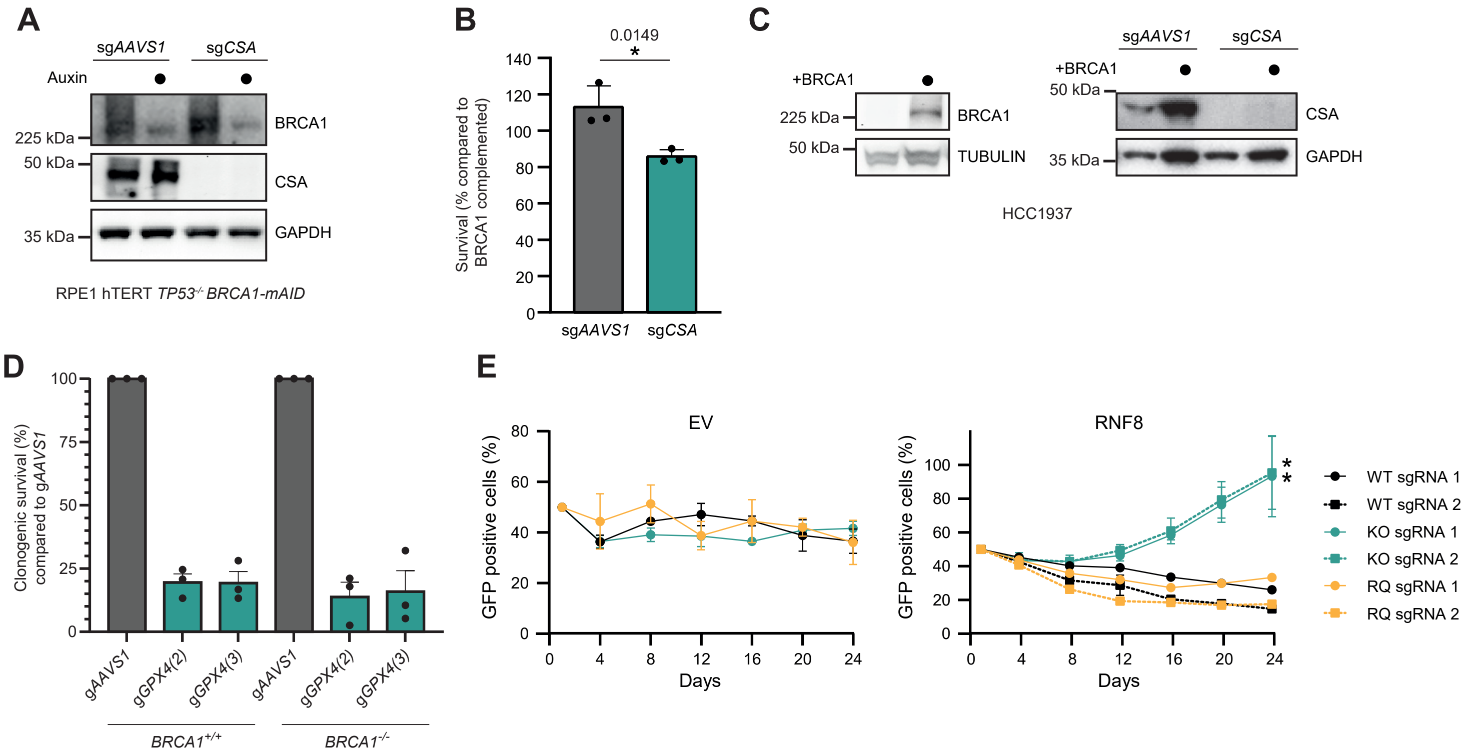

Supplemental figure 2. BRCA1-depleted cells are sensitive to CSA loss (A) Lysates of the RPE1 hTERT *TP53*$^{-/-}$ *BRCA1-mAID* (auxin-inducible degron) cell lines described in Figure 2D were analysed by western blotting. Data shown represent three independent experiments. **(B)** HCC1937 cell lines either complemented with EV or *BRCA1* cDNA, were virally transduced to express *Cas9* cDNA and a sgRNA against *AAVS1* or *CSA*, followed by a MTT viability assay (n=3, mean+SD, *p<0.05, ratio paired t-test). Western blot of lysates shown in Supplemental figure 2C. **(C)** Lysates of the HCC1937 cell lines described in Supplemental figure 2B were analysed by western blotting. Data shown represent three independent experiments. **(D)** Clonogenic survival of RPE1 hTERT *TP53*$^{-/-}$ *BRCA1*$^{+/+}$ or *BRCA1*$^{-/-}$ upon viral transduction of Cas9 and two independent gRNAs targeting *GPX4*. The graph represents the clonogenic survival compared to the survival of the cells transduced with the control gRNA against *AAVS1* (n=3, mean+SD). **(E)** RPE1 hTERT *TP53*$^{-/-}$ *BRCA1-mAID* cells expressing doxycycline inducible Cas9, either BRCA1-proficient (wildtype (WT), black lines), BRCA1-depleted with auxin (knock out (KO), green lines) or BRCA1^{R1699Q} complemented (RQ), orange lines), were infected with indicated *RNF8* sgRNA together with GFP, or with an empty vector together with mCherry. GFP- and mCherry-positive cells were mixed 1:1, and the ratio of GFP-positive cells in the population was determined over time (n=3, mean±SEM, *p<0.05, paired t-test vs WT at t+24). Average eficiency of the sgRNA1 against *RNF8*, determined by using Synthego ICE (Synthego Performance Analysis, ICE Analysis. 2019. v3.0. Synthego) was as follows: 69% in the BRCA1-proficient (WT) cells (including BRCA1-depleted cells after auxin treatment; KO), and 73% in the BRCA1^{R1699Q} complemented cells (RQ).

Supplemental figure 3

A

		Reference (20592)	BRCA1-depleted (213)	BRCA1^{R1699Q} (154)
A	Aberrant regulation of mitotic exit in cancer due to RB1 defects	20	3	0
B	Mitotic Spindle Checkpoint	109	6	12
C	Diseases of mitotic cell cycle	38	4	3
D	Aberrant regulation of mitotic cell cycle due to RB1 defects	36	4	3
E	Recruitment of NuMA to mitotic centrosomes	94	6	7
F	Centrosome maturation	80	6	7
G	Recruitment of mitotic centrosome proteins and complexes	80	6	7
H	Separation of Sister Chromatids	187	9	17
I	Loss of proteins required for interphase microtubule organization from the centrosome	68	6	7
J	Loss of Nlp from mitotic centrosomes	68	6	7
K	Establishment of Sister Chromatid Cohesion	11	4	0
L	Amplification of signal from the kinetochores	92	0	10
M	Amplification of signal from unattached kinetochores via a MAD2 inhibitory signal	92	0	10
N	Resolution of Sister Chromatid Cohesion	123	0	11
O	EML4 and NUDC in mitotic spindle formation	115	0	10

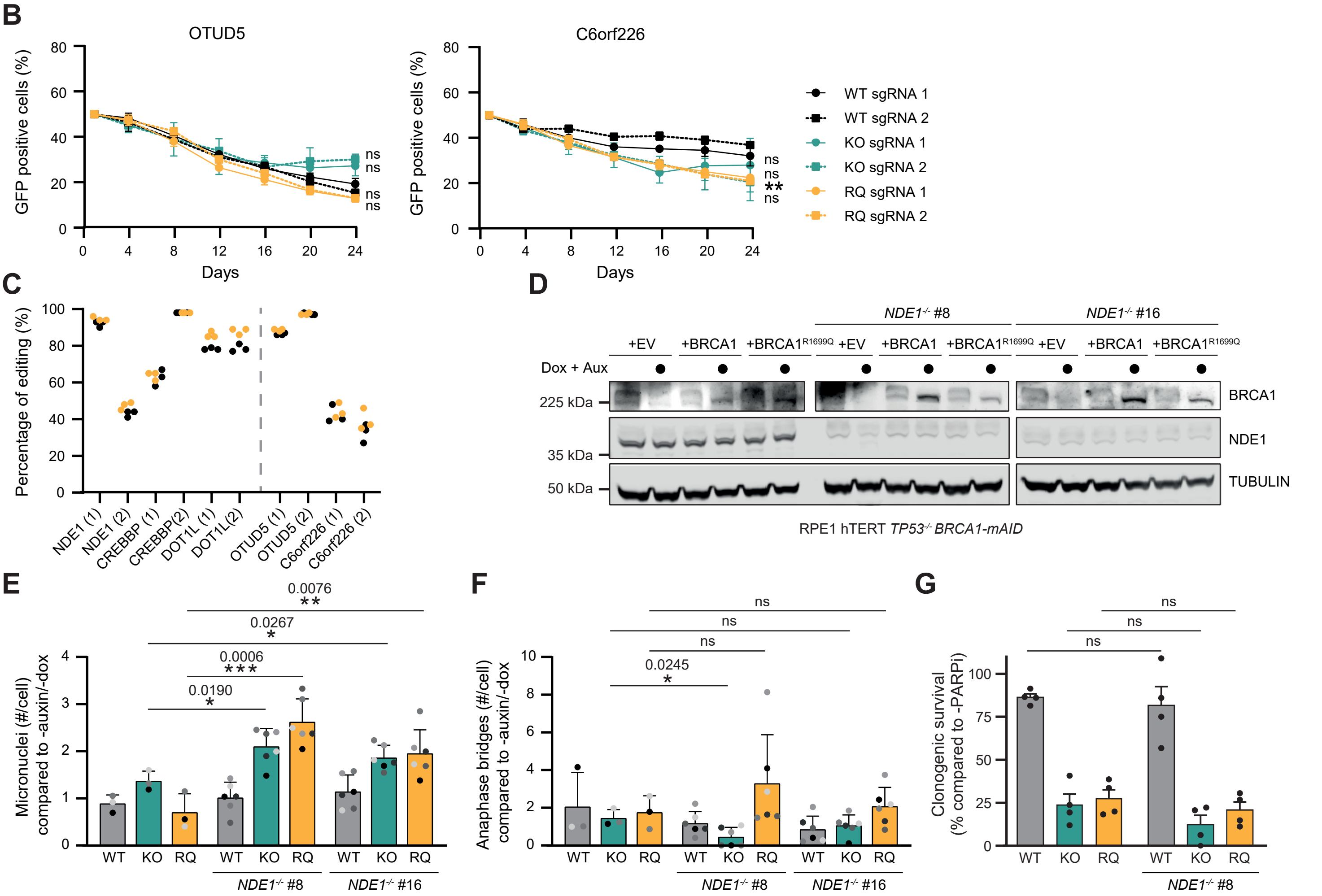

Supplemental figure 3. *NDE1$^{-/-}$* cells were succesfully made and complemented (A) Panther pathway analysis on the top vulnerabilities (NormZ < -2.5) of BRCA1-depleted cells and BRCA1^{R1699Q} cells. Table shows the raw number of genes involved in the specific mitotic process for the two different BRCA1 statuses as shown in Figure 4A. **(B)** RPE1 hTERT *TP53$^{-/-}$ BRCA1-mAID* (auxin-inducible degron) cells expressing doxycycline inducible Cas9, either BRCA1-proficient (wild-type (WT), black lines), BRCA1-depleted with auxin (knock out (KO), green lines) or BRCA1^{R1699Q} complemented (RQ, orange lines), were infected with indicated sgRNA together with GFP, or with an empty vector together with mCherry. GFP- and mCherry-positive cells were mixed 1:1, and the ratio of GFP-positive cells in the population was determined over time (n=3, mean±SEM, ns p>0.05, **p<0.01, paired t-test vs WT at t=24). gRNA efficiency is shown in Supplemental figure 3C. **(C)** Efficiency of each indicated sgRNA per replicate (n=3), determined using Synthego ICE (Synthego Performance Analysis, ICE Analysis. 2019. v3.0. Synthego). Black data points indicate the sgRNA efficiency in BRCA1-proficient (WT) cells (including BRCA1-depleted cells after auxin treatment; KO), orange data points in BRCA1^{R1699Q} complemented cells (RQ). **(D)** Western blot to check the expression of BRCA1 and NDE1 in the lysates decribed in Figure 4C and Supplemental figure 3E,F. Data shown represent three independent experiments. **(E)** The indicated complemented (with either EV, doxycycline inducible BRCA1-WT or BRCA1^{R1699Q}) RPE1 hTERT *TP53$^{-/-}$ BRCA1-mAID NDE1$^{+/+}$* or *NDE1$^{-/-}$* cell lines were grown with or without auxin (to deplete endogenous BRCA1) and doxycycline (to express the BRCA1 complementation) for 48 hours before fixation. Micronuclei per cell were quantified (n=3 for the *TP53$^{-/-}$ BRCA1-mAID NDE1$^{+/+}$* and n=6 for the *TP53$^{-/-}$ BRCA1-mAID NDE1$^{-/-}$*, mean+SD, *p<0.05, **p<0.01, ***p<0.001, unpaired t-test). **(F)** The indicated complemented (with either EV, doxycycline inducible BRCA1-WT or BRCA1^{R1699Q}) RPE1 hTERT *TP53$^{-/-}$ BRCA1-mAID NDE1$^{+/+}$* or *NDE1$^{-/-}$* cell lines were grown with or without auxin (to deplete endogenous BRCA1) and doxycycline (to express the BRCA1 complementation) for 48 hours before fixation. Samples were analysed for anaphase bridges (n=3 for the *TP53$^{-/-}$ BRCA1-mAID NDE1$^{+/+}$* and n=6 for the *TP53$^{-/-}$ BRCA1-mAID NDE1$^{-/-}$*, mean+SD, ns p>0.05, *p<0.05, unpaired t-test). **(G)** The indicated complemented (with either EV, doxycycline inducible BRCA1-WT or BRCA1^{R1699Q}) RPE1 hTERT *TP53$^{-/-}$ BRCA1-mAID NDE1$^{+/+}$* or *NDE1$^{-/-}$* cell lines were treated with auxin (to deplete endogenous BRCA1) and doxycycline (to express the BRCA1 complementation) and with or without 10 nM Olaparib and viability was assessed after 14 days using a clonogenic survival assay (n=4, mean+SEM, ns p>0.05, RM one-way ANOVA, Tukey's multiple comparisons test).
